# Disruption of AKAP-PKA Interaction Induces Hypercontractility With Concomitant Increase in Proliferation Markers in Human Airway Smooth Muscle

**DOI:** 10.3389/fcell.2020.00165

**Published:** 2020-04-09

**Authors:** Hoeke A. Baarsma, Bing Han, Wilfred J. Poppinga, Saskia Driessen, Carolina R. S. Elzinga, Andrew J. Halayko, Herman Meurs, Harm Maarsingh, Martina Schmidt

**Affiliations:** ^1^Department of Molecular Pharmacology, University of Groningen, Groningen, Netherlands; ^2^Groningen Research Institute for Asthma and COPD, University Medical Center Groningen, University of Groningen, Groningen, Netherlands; ^3^Department of Physiology and Pathophysiology, University of Manitoba, Winnipeg, MB, Canada; ^4^Department of Pharmaceutical Sciences, Lloyd L. Gregory School of Pharmacy, Palm Beach Atlantic University, West Palm Beach, FL, United States

**Keywords:** airway smooth muscle, A-kinase anchoring proteins (AKAP), protein kinase A (PKA), asthma, chronic obstructive pulmonary disease (COPD)

## Abstract

With the ability to switch between proliferative and contractile phenotype, airway smooth muscle (ASM) cells can contribute to the progression of airway diseases such as asthma and chronic obstructive pulmonary disease (COPD), in which airway obstruction is associated with ASM hypertrophy and hypercontractility. A-kinase anchoring proteins (AKAPs) have emerged as important regulatory molecules in various tissues, including ASM cells. AKAPs can anchor the regulatory subunits of protein kinase A (PKA), and guide cellular localization via various targeting domains. Here we investigated whether disruption of the AKAP-PKA interaction, by the cell permeable peptide stearated (st)-Ht31, alters human ASM proliferation and contractility. Treatment of human ASM with st-Ht31 enhanced the expression of protein markers associated with cell proliferation in both cultured cells and intact tissue, although this was not accompanied by an increase in cell viability or cell-cycle progression, suggesting that disruption of AKAP-PKA interaction on its own is not sufficient to drive ASM cell proliferation. Strikingly, st-Ht31 enhanced contractile force generation in human ASM tissue with concomitant upregulation of the contractile protein α-sm-actin. This upregulation of α-sm-actin was independent of mRNA stability, transcription or translation, but was dependent on proteasome function, as the proteasome inhibitor MG-132 prevented the st-Ht31 effect. Collectively, the AKAP-PKA interaction appears to regulate markers of the multi-functional capabilities of ASM, and this alter the physiological function, such as contractility, suggesting potential to contribute to the pathophysiology of airway diseases.

## Introduction

Airway smooth muscle (ASM) cells are phenotypic plastic, being able to switch between a more proliferative (and less contractile) or a more contractile (and less proliferative) state in response to mitogens, growth factors (e.g., transforming growth factor-β), and/or alterations in extracellular matrix composition (Halayko et al., 2008). Phenotypic plasticity of ASM plays an important role in the pathophysiology of obstructive pulmonary diseases, such as chronic obstructive pulmonary disease (COPD) and asthma, by enabling airway hypercontractility and ASM hypertrophy and hyperplasia (Halayko et al., 2008), which both contribute to airway narrowing and airflow limitation (Amrani and Panettieri, 2003; Chung, 2005). The hypercontractile phenotype is characterized by an increased expression of contractile proteins, including α-smooth muscle actin (SMA) and calponin (Halayko et al., 2008). We have previously shown that cyclic adenosine monophosphate (cAMP) regulates mitogen-induced phenotypic plasticity (Roscioni et al., 2011a, c). Furthermore, activation of the cAMP effector protein kinase A (PKA) prevents mitogen-induced proliferation by blunting the activation of extracellular regulated kinase 1/2 (ERK1/2) and p70 ribosomal protein S6 kinase (p70S6K), as well as inhibits mitogen-induced reduction in contractile protein expression in ASM strips (Roscioni et al., 2011a, c).

A-kinase anchoring proteins (AKAPs) are scaffolding proteins that provide a molecular platform for other proteins to ensure that signaling effectors are appropriately targeted to different domains, thereby specifying and facilitating intracellular signal transduction (Wong and Scott, 2004). Several AKAP isoforms have been identified that can differ in their cellular localization and interaction partners (Skroblin et al., 2010). A unifying feature of AKAPs is their ability to anchor the regulatory subunits of PKA in proximity to substrates via a conserved short α-helical structure. A cell permeable inhibitor peptide, stearated (st)-Ht31, was developed based on this short α-helical structure to block the interaction between all members of the AKAP family and PKA. St-Ht31 is designed in such a way that it specifically inhibits the interaction between the RII subunits of cAMP-dependent PKA and AKAPs. The sequence of st-Ht31 (24 amino acids) is derived from endogenously expressed, human thyroid RII anchoring protein Ht31 (Pawson and Scott, 1997; Vijayaraghavan et al., 1997; Esseltine and Scott, 2013; Poppinga et al., 2014).

AKAPs are important in compartmentalizing cAMP within the cellular context, thus providing spatio-temporal regulation of the cAMP pathway (Wong and Scott, 2004). Due to the ability of AKAPs to interact with the β2-adrenoceptor and phosphodiesterases, these are important considerations, as β2-agonists and phosphodiesterase inhibitors are commonly used cAMP-elevating drugs for the treatment of respiratory disease (Poppinga et al., 2014).

On this basis, AKAPs regulate a number of cellular responses including intracellular actin dynamics and cell cycle progression (Kim et al., 2013; Han et al., 2015). Cell cycle kinetics is controlled by various effectors, including p70S6K, ERK1/2, proliferating cell nuclear antigen (PCNA), cyclins, and retinoblastoma protein (Rb) (Leonardi et al., 1992; Karpova et al., 1997; Grewe et al., 1999; Bertoli et al., 2013; Dick and Rubin, 2013). Most of these effectors can interact with AKAPs and dysfunction of AKAPs is associated with cell cycle dysregulation (Akakura et al., 2008; Pellagatti et al., 2010; Kong et al., 2013; Han et al., 2015; Petrilli and Fernández-Valle, 2016). For instance, AKAP8 (also known as AKAP95), which is expressed in human ASM, can interact with the cell cycle regulator cyclin D1 (Arsenijevic et al., 2004; Horvat et al., 2012; Poppinga et al., 2015). However, the overall role for AKAPs in regulating cell cycle transition and contractility in human ASM is yet to be defined. Using the specific AKAP-PKA interaction disruptor, st-Ht31 (Skroblin et al., 2010), the current study explores the role of AKAPs in regulating human ASM phenotypic modulation. Here we demonstrate that disruption of AKAP-PKA interactions specifically alters the phosphorylation status or expression of a subset of cell cycle regulatory proteins in human ASM cells, which is unexpectedly paralleled by hypercontractility in human bronchial strips.

## Materials and Methods

### Cell Culture

Human ASM cell cultures were generated from macroscopically healthy 3rd–5th generation bronchial segments obtained from three different donors undergoing lung resection surgery, and thereafter low passage number primary cultures (P2–P3) were made senescence-resistant by stable ectopic expression of human telomerase reverse transcriptase (hTERT) as described previously (Gosens et al., 2006; Burgess et al., 2018). Primary human ASM cells were isolated from human tracheal sections from anonymized lung transplantation donors (obtained from the Department of Cardiothoracic Surgery, University Medical Center Groningen) as previously described (Roscioni et al., 2011b). Cell cultures were maintained in DMEM (Life technologies, 11965-092) containing heat-inactivated fetal bovine serum (10% vol/vol), streptomycin (50 U/ml) and penicillin (50 mg/ml) in a humidified atmosphere at 37°C in air/CO_2_ (95%:5% vol/vol). hTERT ASM cultures up to passage 30 were used.

### [^3^H]-Thymidine Incorporation Assay

hTERT ASM cells were plated in 24-well plates at 20,000 cells/well. After growing to confluence, cells were serum deprived for 3 days and subsequently incubated with 50 μM st-Ht31 (V8211, Promega) or vehicle (i.e., water) for 4 h, then incubated in the presence of [^3^H]-thymidine (0.25 μCi/ml) for 24 h. After incubation, cells were washed twice with PBS at room temperature and subsequently with ice-cold 5% trichloroacetic acid on ice for 30 min and the acid-insoluble fraction was dissolved in 1 ml NaOH (1 M). Incorporated [^3^H]-thymidine was quantified by liquid-scintillation counting using a Beckam LS1701 β-counter as described previously (Roscioni et al., 2011a).

### Cell Viability Assay

hTERT ASM cells were plated in 24-well plates at 20,000 cells/well and serum deprived for 3 days. Subsequently the cells were incubated with st-Ht31 (50 μM) or vehicle for 24 h, after which the cells were washed twice with PBS and incubated with 5% vol/vol AlamarBlue^®^ (DAL1100, Thermo Fisher Scientific) in HBSS for 45 min. AlamarBlue^®^ is converted into its fluorescent form by mitochondrial cytochromes in viable cells. Therefore, the amount of fluorescence is proportional to the number of living cells. Cell viability was assessed by measuring fluorescence emission using a Wallac 1420 Victor 2TM (excitation: 570 nm, emission: 590 nm).

### Fluorescence-Activated Cell Sorting Analysis

To study the effect of st-Ht31 on cell cycle distribution, fluorescence-activated cell sorting (FACS) analysis was performed in the hTERT ASM cells. hTERT ASM cells were plated in 6-well plates at 200,000 cells/well. After growing to confluence, cells were serum deprived for 3 days and subsequently treated with st-Ht31 (50 μM) or vehicle for 24 h. After incubation, cells were detached by trypsin treatment and washed twice with warm PBS (37°C). Cells were then resuspended in ice-cold PBS and subsequently fixed in ice-cold 70% ethanol. After centrifugation, the cell pellet was resuspended in PBS containing 10 μg/ml propidium iodide, 20 mM EDTA, 0.05% Tween 20 and 50 μg/ml RNAse, and incubated overnight at 4°C. Cell cycle analysis was performed on a BD FACSCalibur (Becton, Dickinson and Company, BD, Franklin Lakes, NJ, United States). Fluorescence histograms were collected for at least 10,000 cells. The cell cycle distribution was analyzed using ModFit LT flow cytometry modeling software (ModFit LT, version 4.0.5).

### Western Blot

hTERT ASM cells were plated in 6-well plates at 200,000 cells/well. After serum deprivation for 3 days, cells were treated with st-Ht31 (50 μM) for 24 h. To study the possible mechanisms underlying the effect of st-Ht31 on α-SMA and calponin protein expressions, inhibitors (MG-132: 5 μM, ab141003, Abcam; cycloheximide: 5 mg/ml, 44189, BDH Biochemicals; actinomycin D: 1 μg/ml, A1410, Sigma; chloroquine diphosphate salt: 50 μM, C6628, Sigma) were added for the final 24 h. After washing twice with ice-cold PBS, cells were lysed using 100 μl of RIPA buffer (composition: 50 mM Tris, 150 mM NaCl, 0.1% SDS, 0.5% sodium deoxycholate, 1% nonyl phenoxypolyethoxylethanol), supplemented with 1 mM Na_3_VO_4_, 1 mM NaF, 1,06 mg/ml β-glycerolphosphate, 1 μg/ml apoprotein, 1 μg/ml leupeptin, and 1 μg/ml pepstatin A. The cell lysate was homogenized by passing through a 25-gauge needle for 10 times. Protein content was determined using the Pierce BCA protein assay.

Equal amounts of protein were prepared for SDS-PAGE by adding 4X SDS loading buffer and ultrapure water, and separated on a 10% polyacrylamide gel and transferred to a nitrocellulose membrane, followed by blocking with 1x Roti^®^-Block (A151, Carl Roth), and incubated overnight with primary antibodies (see Table 1). After washing, the membranes were incubated with horseradish peroxidase-labeled secondary antibodies (see Table 1). Protein bands were visualized using Western Lightning^®^ Plus-ECL (NEL104001EA, PerkinElmer) and quantified using ImageJ J 1.48v. Proteins were normalized to GAPDH, lamin A/C, total ERK1/2 (p44/42 MAPK) or caveolin-1, as appropriate.

**TABLE 1:** Antibodies used for Western blot analysis.

Protein of interest	Primary antibody	Secondary antibody
α-sm-actin	1:1000, A2547, Sigma	1:2000, A9044, Sigma
Calponin	1:1000, C2687, Sigma	1:5000, A9044, Sigma
PCNA	1:1000, sc-7907, Santa-Cruz	1:2000, A0545, Sigma
Pan-ubiquitin	1:1000, ab7780, Abcam	1:3000, A0545, Sigma
Cyclin D1	1:1000, #2926, Cell Signaling	1:1000, A9044, Sigma
Phosphorylated (p)-Rb	1:500, #9308, Cell Signaling	1:1000, A0545, Sigma
p-p70S6K	1:500, sc-7984-R, Santa-Cruz	1:2000, A0545, Sigma
p-ERK1/2 (p-p44/42 MAPK)	1:2000, #9101S, Cell Signaling	1:5000, A0545, Sigma
Total ERK1/2 (p44/42 MAPK)	1:2000, #9102, Cell Signaling	1:5000, A0545, Sigma
Caveolin-1	1:1000, sc-894 (HRP conjugated), Santa-Cruz	Directly labeled
Lamin A/C	1:1000, sc-7292, Santa-Cruz	1:2000, A9044, Sigma
GAPDH	1:2000, sc-47724, Santa-Cruz	1:8000, A9044, Sigma

### Immunofluorescence

Primary human ASM cells or immortalized hTERT ASM cells were plated at a density of 20,000 cells per well on a 24-well plate with cover slips placed at the bottom of each well. Cells were serum deprived for 3 days and subsequently treated with st-Ht31 (50 μM) or vehicle for 24 h. After stimulation, cells were washed twice with PBS and fixed with 4% paraformaldehyde + 4% sucrose for 15 min, followed by 0.3% Triton X100 for 2 min at room temperature. Cells were blocked with a blocking buffer containing 5% bovine serum albumin (BSA) and 2% donkey serum at room temperature for 1 h. After fixing, cells were incubated overnight at 4°C with the primary antibody (α-sm-actin: 1:1000, A2547, Sigma; cyclin D1: 1:100, #2926, Cell Signaling; AKAP8: 1:50, sc-10766, Santa-Cruz) diluted in 1% BSA. The next day, after thorough wash, cells were incubated with the secondary antibody (anti-rabbit FITC, Green, 1:500, 65-6111, Thermo Fisher Scientific) for 1 h at room temperature in a dark chamber. After a thorough wash, nuclei were stained with Hoechst (1:10,000, H3570, Invitrogen) for 5–10 s, immediately followed by two quick and four 10 min washing steps with dd-H_2_O. Finally, cover slips were placed and attached on microscope slides using ProLong Gold antifade reagent (Invitrogen). Images were taken and analyzed using an Olympus AX70 microscope equipped with digital image capture system (ColorView Soft System with Olympus U CMAD2 lens, Olympus Corporation, Tokyo, Japan). The background corrected fluorescence measurements were performed with Image J 1.48v as previously described (Burgess et al., 2010).

### mRNA Isolation and Real Time PCR

hTERT ASM cells were plated in 6-well plates at a concentration of 200,000 cells/well. After serum deprivation (1 day for actin alpha 2 (Acta2), calponin 1 (Cnn1), and 3 days for AKAP8), cells were treated with st-Ht31 (50 μM) for 10 h. To determine the half-life of ACTA2 mRNA, hTERT ASM cells were pretreated with Actinomycin D (4 μM) 30 min prior to treatment with st-Ht31 (50 μM) or vehicle for various time-points up to 24 h. mRNA from hTERT ASM cells was extracted using a Nucleospin RNA II kit (Machery Nagel) and quantified using spectrophotometry (NanoDrop, Thermo Fisher Scientific). 1 μg of mRNA was converted into cDNA by reverse transcriptase using Promega tools (Madison). cDNA was subjected to real-time PCR (RT-PCR) using a MyiQ^TM^ Single-Color detection system (Bio-Rad Laboratories Inc. Life Science Group) and the specific primers (see Table 2). RT-qPCR was performed in duplicate using SYBR Green (Roche) with denaturation at 94°C for 30 s, annealing at 59°C for 30 s and extension at 72°C for 30 s for 40 cycles followed by 10 min at 72°C. The amount of target gene was normalized to ribosomal subunit 18 S (designated as ΔCT). Relative differences were determined using the equation 2^–(ΔΔCt)^.

**TABLE 2:** primers used for qRT-PCR.

Gene of interest	Primers
Acta2	Forward 5′-CTTTCATTGGGATGGAGTCAGC-3′
	Reverse 5′-ACAGGACGTTGTTAGCATAGAGA-3′
Cnn2	Forward 5′-TCTTTGAGGCCAACGACCTG-3′
	Reverse 5′-GGGATCATAGAGGTGACGCC-3′
AKAP8	Forward 5′-ATGCACCGACAATTCCGACT-3′
	Reverse 5′-CATAGGACTCGAACGGCTGG-3′
18S	Forward 5′-CGCCGCTAGAGGTGAAATTC-3′
	Reverse 5′-TTGGCAAATGCTTTCGCTC-3′

### Human Tracheal Smooth Muscle Strips

Human tracheal tissue from anonymized lung transplantation donors was obtained from the Department of Cardiothoracic Surgery, University Medical Center Groningen. All tissue was collected according to the Research Code of the University Medical Center Groningen^1^ and national ethical and professional guidelines (“Code of conduct,” Dutch federation of biomedical scientific societies^2^). After dissection of the smooth muscle layer and careful removal of the mucosa and connective tissue, human tracheal smooth muscle strips of identical length and width were prepared as described previously (Roscioni et al., 2011c). The average weight of the bronchial strips is 13.9 ± 5.9 mg (average ± standard deviation, *n* = 55). The main difference in weight is due to interindividual differences between tissue of the donors, rather than between bronchial strips derived from the same donor. For each experiment, we randomize the prepared bronchial strips before subsequent treatment is started. To limit the possibility of variations between tissue preparations of the same donor, we perform each experiment at least in duplicate and the average value for the contractility of both tissue strips together is considered as one independent data-point. Tissue strips were transferred to serum-free DMEM supplemented with sodium pyruvate (1 mM), non-essential amino acid mixture (1:100), gentamicin (45 μg/ml), penicillin (100 U/ml), streptomycin (100 μg/ml), amphotericin B (1.5 μg/ml), apo-transferrin (human, 5 μg/ml) and ascorbic acid (100 μM). The strips were incubated with st-Ht31 (50 μM) or vehicle for 24 h in an Innova 4000 incubator shaker (37°C, 55 rpm). After culture, strips were thoroughly washed and mounted in an organ bath for isometric tension measurements.

### Isometric Contraction Measurement

Isometric contraction experiments were performed essentially as described previously (Roscioni et al., 2011c). Briefly, ASM strips were mounted for isometric recording in 20 ml organ-baths, containing Krebs-Henseleit (composition in mM: NaCl 117.5, KCl 5.60, MgSO_4_ 1.18, CaCl_2_ 2.50, NaH_2_PO_4_ 1.28, NaHCO_3_ 25.00, and glucose 5.50) buffer at 37°C. During a 90 min equilibration period with wash-outs every 30 min, resting tension was adjusted to 1 g, followed by pre-contractions with 10 μM methacholine. Following wash-out, maximal relaxation was established by the addition of 0.1 μM (-)-isoprenaline. Tension was readjusted to 1 g, followed by refreshing of the Krebs-Henseleit buffer twice. After another equilibration period of 30 min, cumulative concentration–response curves were constructed with methacholine (0.1 nM – 1 mM). When maximal tension was reached, strips were washed several times and maximal relaxation was established using 10 μM (-)-isoproterenol. Contractions were corrected for tissue weight and expressed as percentage of the maximal methacholine-induced contraction in vehicle-treated strips. Curves were fitted using Prism 5.0. After the contraction protocol, strips were collected and tissue homogenates were prepared as previously described (Roscioni et al., 2011c) for western blot measurement of α-sm-actin, calponin and PCNA.

### Statistics

Data are expressed as means ± SEM of *n* individual experiments. Statistical significance of differences was evaluated using Prism 5.0 software by performing One-sample *T*-tests. For the isometric contraction experiments: non-linear curve fit was performed and subsequent statistical curve comparison was done using the extra sum-of-squares *F*-test using Prism 5.0 software. Differences were considered to be statistically significant when *p* < 0.05.

## Results

### Role of AKAPs in Proliferation of Human ASM Cells

**FIGURE 1:**
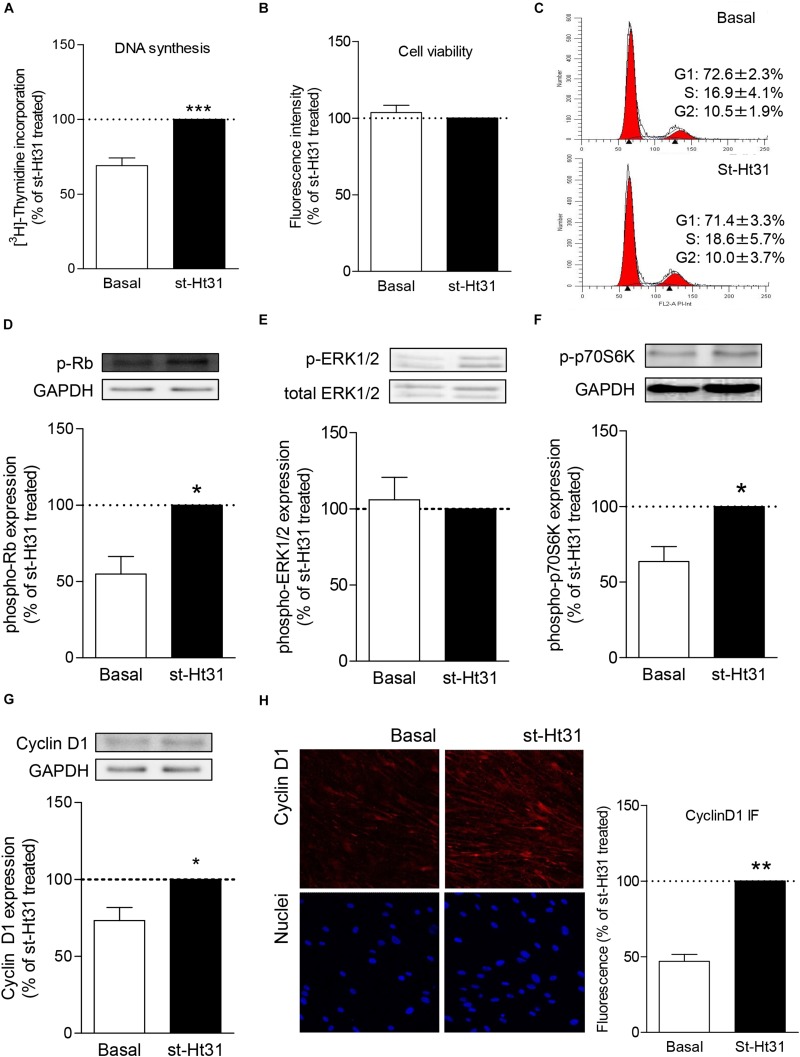
The effects of st-Ht31 on proliferation markers in human airway smooth muscle cells. hTERT ASM cells were serum-deprived for 3 days and treated with st-Ht31 (50 μM). **(A)** [^3^H]-thymidine was added 4h after st-Ht31 and incorporated [^3^H]-thymidine was quantified 2 4h later. *n* = 15. **(B)** After 24h of treatment with st-Ht31, cell viability was assessed using AlamarBlue^®^. *n* = 5. **(C)** FACS analysis was performed 24 h after st-Ht31 treatment. *n* = 3. **(D–H)** Protein expression of the indicated proteins was measured 24 h after st-Ht31 treatment using Western blot **(D–G)** or immunofluorescence (IF, **H**). *n* = 4–9. *p < 0.05, **p < 0.05, and ***p < 0.001 compared to basal.

Treatment with st-Ht31 significantly increased [^3^H]-thymidine incorporation in hTERT ASM cells (Figure 1A), indicating enhanced DNA synthesis. However, st-Ht31 treatment for 4 days did not affect cell viability (Figure 1B). We further assessed cell cycle distribution of propidium iodide stained hTERT ASM cells by flow cytometry and found that st-Ht31 exposure had little effect (Figure 1C).

To further understand the paradoxical increase in DNA synthesis without cell cycle induction with st-Ht31 treatment, we investigated the abundance and phosphorylation status of cell cycle regulator proteins. Treatment of hTERT ASM cells with st-Ht31 enhanced the phosphorylation status of the cell-cycle regulators Rb and p70S60K, without affecting ERK1/2 (p42/44 MAPK) phosphorylation (Figures 1D–F). Moreover, st-Ht31 treatment increased cyclin D1 abundance in hTERT ASM cells (Figures 1G,H).

### AKAP8 Expression and Localization in Human ASM Cells

**FIGURE 2:**
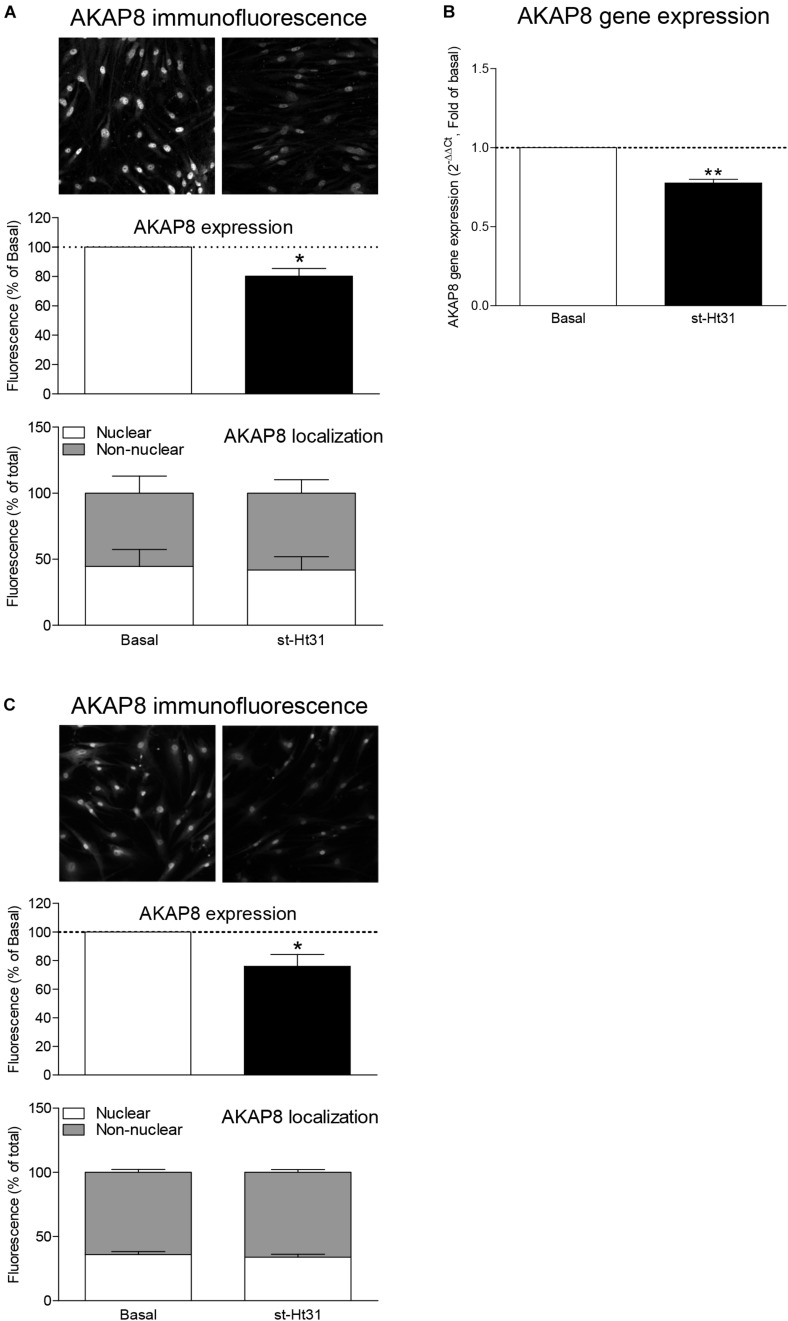
The effects of st-Ht31 on AKAP8 expression and cellular localization in human airway smooth muscle cells. **(A)** hTERT ASM cells were serum deprived for 3 days and treated with st-Ht31 (50 μM) for 24 h. The expression and localization of AKAP8 was measured by immunofluorescence. Representative images are shown. Images were quantified by Image J 1.48v. *n* = 4. **(B)** AKAP8 mRNA expression was measured in hTERT ASM cells using RT-PCR and normalized to ribosomal subunit 18 S (ΔCT). Relative differences were determined using the equation 2^–(ΔΔCt)^. *n* = 4. **(C)** Primary human ASM cells were serum deprived for 3 days and treated with st-Ht31 (50 μM) for 24 h. The expression and localization of AKAP8 were measured by immunofluorescence. Representative images are shown. Images were quantified by Image J 1.48v. *N* = 5, *p < 0.05 and **p < 0.01 compared to basal.

Previous studies by us and others demonstrated protein and/or mRNA expression of a subset of AKAPs in human ASM, including AKAP1, AKAP2, AKAP3, AKAP5 (also known as AKAP79), AKAP8, AKAP9, AKAP10, AKAP11, AKAP12, AKAP13, Ezrin, and MAP2B (Horvat et al., 2012; Poppinga et al., 2015). We currently investigated the effect of st-Ht31 on the expression of AKAP8, as this particular AKAP is associated with cell cycle progression (Han et al., 2015). Treatment of hTERT ASM cells with st-Ht31 decreased the fluorescent intensity of total AKAP8 compared to vehicle treated cells (Figure 2A). The ratio of the cellular distribution of AKAP8 between nuclei and non-nuclear compartments was not altered by st-Ht31, indicating that st-Ht31 causes a general decrease in AKAP8 abundance. In line with the changes in protein level, we found that st-Ht31 also significantly decreased AKAP8 mRNA expression (Figure 2B). Besides AKAP8, we also measured the expression of AKAP5, AKAP12, and Ezrin, but none were significantly altered by st-Ht31 (data not shown). Primary human ASM cells also showed decreased AKAP8 expression, without an effect on its cellular distribution, in response to st-Ht31 (Figure 2C).

### Role of AKAPs in Contractile Protein Expression in Human ASM Cells

**FIGURE 3:**
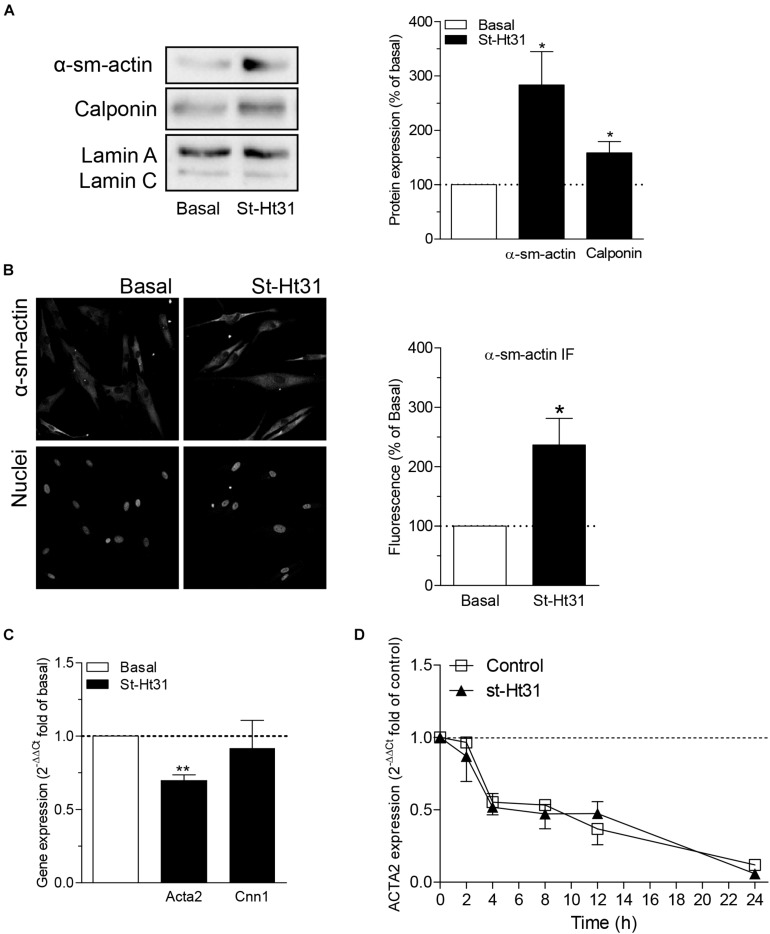
The effect of st-Ht3 on contractile markers in human airway smooth muscle cells. **(A,B)** hTERT ASM cells were serum deprived for 3 days and treated with st-Ht31 (50 μM) for 24 h. Protein expression of α-sm-actin **(A,B)** and calponin **(A)** were measured by western blot **(A)** or immunofluorescence (IF, **B**). Data expressed as means ± SEM of *n* = 5–8. **(C)** hTERT ASM cells were serum deprived for 1 day and treated with st-Ht31 (50 μM) for 1 h. *ACTA2* and *CNN1* expression was measured using RT-PCR and normalized to ribosomal subunit 18 S (ΔCT). Relative differences were determined using the equation 2^–(ΔΔCt)^. *n* = 4. **(D)** hTERT ASM cells were pretreated with actinomycin D (4 μM) and subsequently treated without (control) or with st-Ht31 (50 μM) for the indicated time-points. *ACTA2* expression was measured using RT-PCR and normalized to ribosomal subunit 18 S (ΔCT). Relative differences were determined using the equation 2^–(ΔΔCt)^. *n* = 6. *p < 0.05 and **p < 0.01 compared to basal.

Treatment of hTERT ASM cells with st-Ht31 resulted in a significant increase in the abundance of the contractile proteins α-sm-actin and calponin (Figures 3A,B). Remarkably, the mRNA abundance for *ACTA2* and *CNN1* (encoding α-sm-actin and calponin, respectively) was decreased (i.e., *ACTA2*) or unaltered (i.e., *CNN1*) in response to st-Ht31 (Figure 3C). Next, we investigated if disruption of the AKAP-PKA interaction in human ASM cells by st-Ht31 affected mRNA stability of *ACTA2*. Expression of *ACTA2* decreased over time in human ASM cells and this process was unaffected by st-Ht31 (Figure 3D).

**FIGURE 4:**
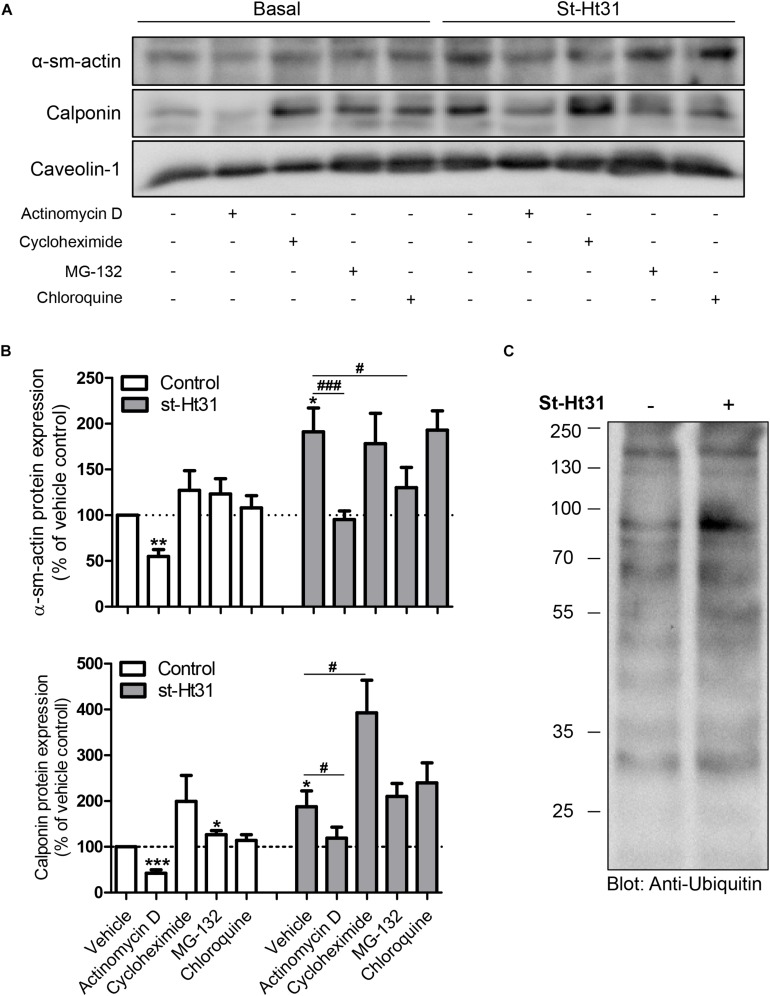
Potential mechanisms underlying st-Ht31-induced contractile protein expression in human airway smooth muscle cells. **(A,B)** hTERT ASM cells were treated with st-Ht31 (50 μM) in the absence or presence of actinomycin D (1 μg/ml), cycloheximide (5 mg/ml), MG-132 (5 μM) and chloroquine (50 μM). Protein expression of α-sm-actin and calponin was analyzed and quantified. *n* = 5–8 experiments. **(C)** hTERT ASM cells were treated with vehicle or st-Ht31 (50 μM) for 1h. The degree of protein ubiquitination in whole cell lysate was detected using an anti-ubiquitin antibody. *p < 0.05, **p < 0.01, and ***p < 0.001 compared to vehicle without st-Ht31, ^#^*p* < 0.05 and ^###^*p* < 0.001 compared to vehicle with st-Ht31.

To investigate the potential mechanism(s) for the differential effects of st-Ht31 on mRNA and protein abundance of contractile proteins α-sm-actin and calponin, we independently inhibited gene transcription, translation, and protein degradation using pharmacological tools. Inhibition of RNA synthesis by actinomycin D decreased basal and st-Ht31-induced α-sm-actin and calponin protein abundance in human ASM cells (Figures 4A,B). However, compared directly to time-matched actinomycin D treated cultures, the addition of st-Ht31 still increased sm-α-actin and calponin abundance in human ASM cells (Figures 4A,B). Cycloheximide, an inhibitor of protein synthesis, did not affect (α-sm-actin) or even enhanced (calponin) contractile protein abundance in the presence or absence of st-Ht31 (Figures 4A,B). Pharmacological suppression of protein degradation with the proteasome inhibitor MG-132, was without major effect on basal abundance of sm-α-actin and calponin, but prevented the induction of α-sm-actin by st-Ht31 (Figures 4A,B). Concomitant, we observed a transient increase in ubiquitination of a variety of proteins in response to short-term st-Ht31 treatment (Figure 4C). Finally, inhibition of protein turnover by targeting lysosomal enzymes with chloroquine did not affect contractile protein abundance at baseline or after treatment with st-Ht31 (Figures 4A,B).

### Role of AKAPs in Regulating Contractility and Proliferation in Human Bronchial Strips

**FIGURE 5:**
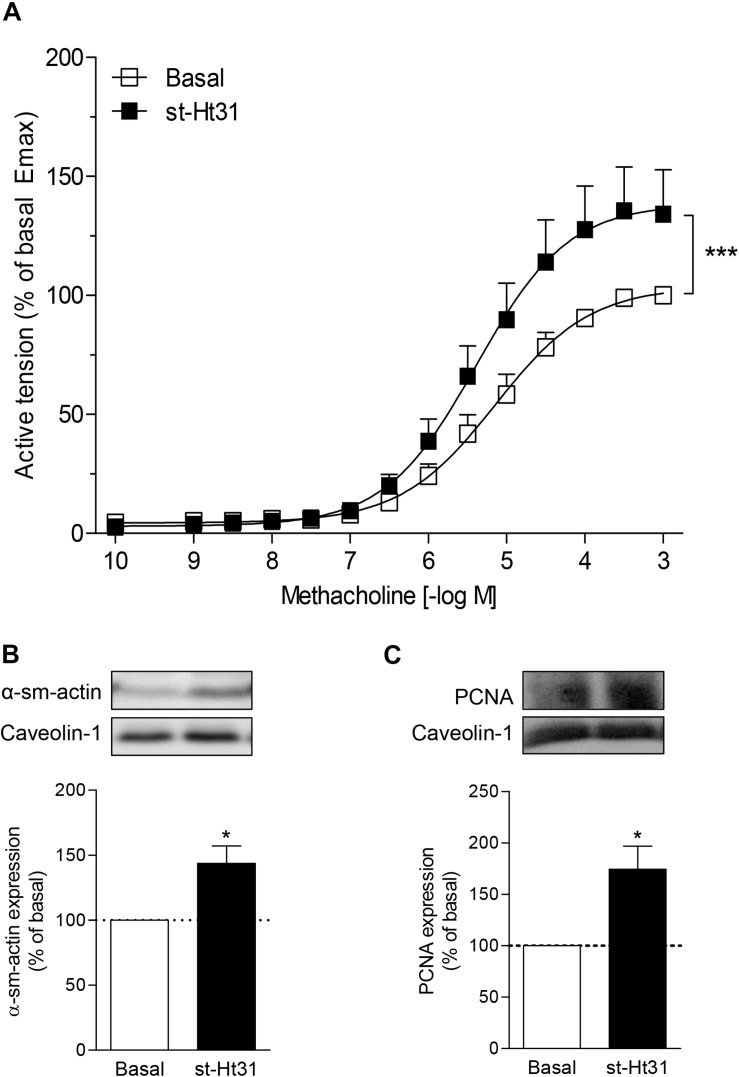
The effects of st-Ht31 on contraction of human airway smooth muscle strips. Isolated human tracheal smooth muscle strips were incubated without or with st-Ht31 (50 μM). **(A)** Methacholine-induced isometric contraction was measured. *N* = 7–8. ***p < 0.001 compared to basal as determined by an extra sum-of-squares *F*-test. **(B,C)** The expression of α-sm-actin **(B)** and PCNA **(C)** was determined in the tracheal strips by immunoblotting. *N* = 4–5, *p < 0.05 compared to basal.

To investigate the functional consequence of increased contractile protein abundance that we observed in cultured ASM cells, we incubated human ASM tissue strips with st-Ht31 and subsequently assessed methacholine-induced isometric contraction. Treatment with st-Ht31 significantly increased contraction by 1.3-fold (E_max_; Figure 5A), without affecting the sensitivity to methacholine (pD_2_-values: 5.28 ± 0.18 and 5.36 ± 0.18 for basal and st-Ht31 treated, respectively). The increased capacity to generate (maximum) force was accompanied by a concomitant increase in α-sm-actin protein abundance (Figure 5B), whereas calponin expression showed more variation and was not significantly altered (data not shown). Furthermore, treatment of human ASM strips with st-Ht31 resulted in an increase in the S-phase marker *proliferating cell nuclear antigen* (PCNA) (Figure 5C).

## Discussion

We demonstrate that disruption of AKAP-PKA interactions using st-Ht31 increases expression of the contractile proteins α-sm-actin and calponin in human ASM cells, and development of hypercontractile human ASM in bronchial strips. In parallel, st-Ht31 enhances DNA synthesis and expression of markers for ASM cell proliferation, although neither cell viability nor cell cycle distribution are affected directly. Collectively, these findings indicate that AKAP-PKA mediated signaling regulates ASM phenotype and function.

AKAPs are a group of structurally diverse proteins, which act as scaffolds for a variety of structural and signaling molecules to facilitate targeting of different cellular microdomains (Wong and Scott, 2004; Poppinga et al., 2014). We recently demonstrated that the expression of several AKAPs in ASM and bronchial epithelial cells is differentially affected by cigarette smoke (Oldenburger et al., 2014; Poppinga et al., 2015). Moreover, we observed differential expression of AKAPs in lung tissue of COPD patients (Oldenburger et al., 2014; Poppinga et al., 2015). All AKAPs combine with the regulatory subunits of PKA through a short α-helical structure to guide activity in different sub-cellular locales (Esseltine and Scott, 2013). This functional feature was exploited in the development of the st-Ht31 peptide, as it mimics the short α helical structure in AKAPs, to competitively block AKAP interaction with PKA (Vijayaraghavan et al., 1997). However, stimulus-induced PKA activation in human ASM is not affected by st-Ht31 (Poppinga et al., 2015). Since its development, st-Ht31 has been shown to alter a variety of cellular functions, including cardiac muscle contraction and cell cycle progression (McConnell et al., 2009; Gao et al., 2012).

ASM cells exhibit phenotype plasticity, which allows modulation of contractile and proliferative functional capacity in response to changes in the surrounding microenvironment (Halayko et al., 2008). Prolonged exposure to mitogens induces a hyperproliferative, hypocontractile ASM phenotype, characterized by increased DNA synthesis and mitosis, as well as reduced contractile protein abundance (Halayko et al., 2008; Simeone-Penney et al., 2008; Roscioni et al., 2011a, c). On the other hand, other stimuli such as transforming growth factor-β1 (TGF-β1), insulin or changes in extracellular laminin composition can induce a hypoproliferative, hypercontractile ASM phenotype (Ma et al., 1998; Gosens et al., 2003; Schaafsma et al., 2007; Halayko et al., 2008; Dekkers et al., 2009; Tran et al., 2013, Ojiaku et al., 2018). Interestingly, we now demonstrate that interrupting the interaction between PKA and AKAPs simultaneously increases markers of a hyperproliferative (e.g., DNA synthesis, activation of cell cycle proteins and increased expression of the proliferative marker PCNA) and a hypercontractile phenotype (e.g., increased expression of contractile proteins and increased contractility) in cultured human ASM cells and in intact human ASM strips.

A potential modulator of ASM phenotype is p70 ribosomal S6 kinase (p70S6K) (Halayko et al., 2004; Roscioni et al., 2011a). Mitogen exposure of human ASM cells leads to phosphorylation and activation of p70 ribosomal S6 kinase (p70S6K), which promotes cell proliferation via upregulation of various proteins, including the cell-cycle checkpoint determinant cyclin D1 (Scott et al., 1996; Karpova et al., 1997; Grewe et al., 1999; Takuwa et al., 1999; Ravenhall et al., 2000; Chambard et al., 2007; Roscioni et al., 2011b). We have previously demonstrated that p70S6K phosphorylation reduced by activation of PKA (Roscioni et al., 2011a). Disruption of the AKAP-PKA interaction by st-Ht31 leads to a loss of intracellular targeting of PKA, which, however, is not accompanied by a loss of PKA activity (Poppinga et al., 2015). Although PKA can still be activated, it cannot signal properly in a spatiotemporal manner in the presence of st-Ht31. In the current study, we observed an enhanced p70S6K phosphorylation and increased Cyclin D1 expression in human ASM in response to st-Ht31, without an effect on ERK1/2 activity. Cyclin D1 associates with pre-existing cyclin-dependent kinases to phosphorylate target proteins, such as Rb, that further enable and modulate cell cycle progress into S phase (Lundberg and Weinberg, 1998). In agreement, st-HT31 concomitantly increases phosphorylation of Rb and DNA synthesis (Withers et al., 1997; Takuwa et al., 1999). Moreover, in intact human bronchial strips st-Ht31 treatment resulted in increased PCNA expression, a protein that is expressed during the S phase of the cell cycle (Leonardi et al., 1992). Nonetheless, the st-HT31-induced DNA synthesis was not accompanied by a change in cell cycle distribution nor by an alteration of cell viability, suggesting that disruption of the AKAP-PKA interaction is not sufficient on its own to induce cells to traverse the S phase of the cell cycle. Our FACS analysis in human ASM cells confirm that st-Ht31 has no significant impact on the fraction of cells in the S phase. Interestingly, AKAPs, particularly AKAP8, interact with cyclins, including cyclin D1 (Eide et al., 1998; Arsenijevic et al., 2004; Han et al., 2015; Qi et al., 2015). AKAP8 can reside in the nucleus, as confirmed by our study, and is thought to be involved in DNA replication and expression of several genes associated with cell cycle regulation (Coghlan et al., 1994; Eide et al., 1998; Han et al., 2015). An AKAP8-cyclin D binding site has been identified that overlaps with a CDK4 binding site, suggesting that nuclear AKAP8 may compete with CDK4 for cyclin D1 (Arsenijevic et al., 2006). Therefore, the observed downregulation of AKAP8 by st-Ht31 in ASM cells, without alterations in cellular distribution, could be permissive for interaction of cyclin D1 with CDK4, thereby supporting Rb phosphorylation and early S phase activities. On the other hand, AKAP8 has been found to regulate M phase events of the cell cycle, such as chromatin condensation, by interacting with DNA and associated proteins including a condensin complex component, Eg7 and histone deacetylase 3 (Collas et al., 1999; Steen et al., 2000; Li et al., 2006). Based on this, and in light of our observations, we hypothesize that, in addition to disrupting the interactions between AKAPs and PKA, st-Ht31-induced AKAP8 downregulation could contribute to disturbed cell cycle kinetics, that in our studies are revealed by increased proliferative markers without an increase in cell number.

Another interesting observation from our study is that disruption of the AKAP-PKA interaction by st-HT31 increases contractile protein expression in human ASM. However, st-Ht31 had little or no effect on CNN1 expression and even decreased ACTA2 expression. Our results also demonstrate that st-Ht31 does not affect mRNA stability of ACTA2. Furthermore, blocking of RNA synthesis by actinomycin D decreased both basal and st-HT31-induced protein abundance of α-sm-actin and calponin. However, compared to solely actinomycin D treatment addition of st-Ht31 still induced an increase in contractile protein expression in human ASM cell. This suggests that the effects of st-Ht31 are, in part, due to post-translational effects. This is confirmed by our observation that inhibition of protein translation using cycloheximide did not affect st-Ht31-induced contractile protein accumulation. Recently, AKAPs have been identified as factors involved in protein turnover, specifically involved with ubiquitin-proteasome systems that tag substrate proteins for subsequent degradation by the covalent attachment of ubiquitin (Rinaldi et al., 2015). This system involves modification of substrate proteins by the covalent attachment of multiple ubiquitin molecules. Ubiquitination is a post-translational modification that generally directs proteins for degradation by the proteasome. However, ubiquitination has also been implicated in many other cellular processes, including transcriptional regulation (Ciechanover, 2005; Stringer and Piper, 2011). We show that treatment of human ASM with the proteasome inhibitor MG-132 prevents st-Ht31-induced α-sm-actin accumulation. In agreement, in vascular smooth muscle cells MG-132 reduces contractile protein expression by reducing myocardin activation (Yin et al., 2011). Myocardin and myocardin related transcription factors (MRTFs) are transcriptional co-activators of serum response factor (SRF), a master regulator of α-smooth muscle actin. In cardiac myofibroblasts, α-sm-actin expression is linked to AKAP-Lbc (also known as AKAP13) by its regulatory effects on myocardin related transcription factors (MRTFs) (Cavin et al., 2014). Clearly, future studies are necessary to investigate the effects of st-HT31 as a potential regulator of myocardin and/or MRTF activation and subsequent contractile protein expression in human ASM cells.

Most importantly, our functional analyses demonstrated increased contraction of human bronchial strips after st-Ht31 treatment. This hypercontractile ASM phenotype is accompanied by increased abundance of α-sm-actin. Of note, the involvement of AKAPs in muscle contraction has previously been shown in the heart (McConnell et al., 2009). Similar to the findings in cultured cells, st-Ht31 also increased a marker of proliferation, i.e., PCNA, in *ex vivo* human bronchial strips. Thus, markers of proliferation and contractility are simultaneously increased in ASM tissue and cultured cells. However, st-Ht31 does not alter cell viability in cultured cells, but does increase contractility of intact human ASM tissue. These findings demonstrate the importance of studying functional readouts for the different phenotypes: cell proliferation and contractility. Thus, st-Ht31 may initially induce markers that could underpin modulation to a proliferative phenotype. However, since it is insufficient to fully induce cell cycle progression and proliferation, this appears to trigger a response, either indirectly or in a mutually exclusive manner, that leads to a later induction of a hypercontractile phenotype. This hypothesis is supported by the finding that insulin acutely induces DNA synthesis in cultured ASM cells, but long-term treatment with insulin induces a hypercontractile phenotype in intact ASM tissue (Gosens et al., 2003).

In summary, we show that st-Ht31 leads to a simultaneous increase in markers of a hypercontractile phenotype and those of a hyperproliferative phenotype in both ASM cells and intact ASM strips. However, increased DNA synthesis and the activation of early cell cycle regulators are not sufficient to complete proliferation and increase cell number. In contrast, the increase in contractile protein expression (marker of a hypercontractile phenotype) did lead to an increase in contractility, indicating the overall effect of disruption of AKAP-PKA interaction is the induction of a hypercontractile phenotype. Previous studies from our group and others, have reported on the expression of at least 12 AKAP family members in human ASM, which might be of interest to target independently or all together by silencing and/or Crisp/Cas9 technologies in future studies (Horvat et al., 2012; Poppinga et al., 2015). In conclusion, AKAP-PKA interactions in ASM cells and tissue limits the contractile function of ASM, likely by restricting the expression of contractile proteins and the development of a hyperproliferative phenotype. Our observations have biological and drug development implications in obstructive respiratory disease, such as asthma and COPD, where there is both an increase in ASM mass and ASM contractility (Lambert et al., 1993; Chung, 2005, 2008; Bentley and Hershenson, 2008). The mechanisms of drugs used in the treatment of respiratory disease, such as β_2_-agonists and phosphodiesterase inhibitors, most likely encompass proper AKAP-PKA interactions.

## Data Availability Statement

The datasets generated for this study are available on request to the corresponding author.

## Ethics Statement

All tissue was collected according to the Research Code of the University Medical Center Groningen (https://www.umcg.nl/SiteCollectionDocuments/English/Researchcode/UMCG-Researchcode,%20basic%20principles%202013.pdf) and national ethical and professional guidelines (“Code of conduct,” Dutch federation of biomedical scientific societies, http://www.federa.org). The patients/participants provided their written informed consent to participate in this study.

## Author Contributions

BH, WP, SD, and CE designed and performed the experiments and performed the data analyses. HB, HMe, HMa, and MS designed the experiments and oversaw all data analyses. AH provided immortalized human airway smooth muscle cells. HB, BH, and MS drafted the figures and manuscript. All authors have critically revised the manuscript, reviewed, and approved the final manuscript as submitted to take public responsibility for it.

## Conflict of Interest

The authors declare that the research was conducted in the absence of any commercial or financial relationships that could be construed as a potential conflict of interest.

## Footnotes

1 https://www.umcg.nl/SiteCollectionDocuments/Englis/Researchcode/UMCG-Researchcode,%20basic%20principles%202013.pdfh

2 http://www.federa.org
